# Projections of Ebola outbreak size and duration with and without vaccine use in Équateur, Democratic Republic of Congo, as of May 27, 2018

**DOI:** 10.1371/journal.pone.0213190

**Published:** 2019-03-07

**Authors:** J. Daniel Kelly, Lee Worden, S. Rae Wannier, Nicole A. Hoff, Patrick Mukadi, Cyrus Sinai, Sarah Ackley, Xianyun Chen, Daozhou Gao, Bernice Selo, Mathais Mossoko, Emile Okitolonda-Wemakoy, Eugene T. Richardson, George W. Rutherford, Thomas M. Lietman, Jean Jacques Muyembe-Tamfum, Anne W. Rimoin, Travis C. Porco

**Affiliations:** 1 School of Medicine, University of California, San Francisco (UCSF), San Francisco, CA, United States of America; 2 F.I. Proctor Foundation, UCSF, San Francisco, CA, United States of America; 3 School of Public Health, University of California, Los Angeles, Los Angeles, CA, United States of America; 4 National Institute of Biomedical Research, Kinshasa, Democratic Republic of Congo; 5 Mathematics and Science College, Shanghai Normal University, Shanghai, China; 6 Ministry of Health, Kinshasa, Democratic Republic of Congo; 7 School of Public Health, University of Kinshasa, Kinshasa, Democratic Republic of Congo; 8 Harvard Medical School, Boston, MA, United States of America; 9 Brigham and Women’s Hospital, Boston, MA, United States of America; Tulane University, UNITED STATES

## Abstract

As of May 27, 2018, 6 suspected, 13 probable and 35 confirmed cases of Ebola virus disease (EVD) had been reported in Équateur Province, Democratic Republic of Congo. We used reported case counts and time series from prior outbreaks to estimate the total outbreak size and duration with and without vaccine use. We modeled Ebola virus transmission using a stochastic branching process model that included reproduction numbers from past Ebola outbreaks and a particle filtering method to generate a probabilistic projection of the outbreak size and duration conditioned on its reported trajectory to date; modeled using high (62%), low (44%), and zero (0%) estimates of vaccination coverage (after deployment). Additionally, we used the time series for 18 prior Ebola outbreaks from 1976 to 2016 to parameterize the Thiel-Sen regression model predicting the outbreak size from the number of observed cases from April 4 to May 27. We used these techniques on probable and confirmed case counts with and without inclusion of suspected cases. Probabilistic projections were scored against the actual outbreak size of 54 EVD cases, using a log-likelihood score. With the stochastic model, using high, low, and zero estimates of vaccination coverage, the median outbreak sizes for probable and confirmed cases were 82 cases (95% prediction interval [PI]: 55, 156), 104 cases (95% PI: 58, 271), and 213 cases (95% PI: 64, 1450), respectively. With the Thiel-Sen regression model, the median outbreak size was estimated to be 65.0 probable and confirmed cases (95% PI: 48.8, 119.7). Among our three mathematical models, the stochastic model with suspected cases and high vaccine coverage predicted total outbreak sizes closest to the true outcome. Relatively simple mathematical models updated in real time may inform outbreak response teams with projections of total outbreak size and duration.

## Introduction

On May 8, 2018, the World Health Organization (WHO) announced the occurrence of an outbreak of Ebola virus disease (EVD) in the Democratic Republic of Congo (DRC).[1] From April 4 through May 7, 21 suspected EVD cases were reported in Iboko and Bikoro, Équateur Province. On May 7, blood samples from five hospitalized patients had been sent to Kinshasa for Ebola-PCR testing, and two were confirmed PCR-positive.[2] On May 21, vaccination of healthcare workers started.[3] By May 27, the ring vaccination campaign was being rolled out as 906 contacts and contacts of contacts were being actively monitored. Six suspected, 13 probable and 35 confirmed EVD cases had been reported, and 25 (52%) of 48 probable and confirmed EVD cases had died.[1]

This outbreak had several features that were worrisome for widespread transmission. Cases were reported over a 168-kilometer distance, including four confirmed cases in the ~1,200,000-inhabitant provincial capital of Equateur, Mbandaka, which is situated on the Congo River and bordering Congo-Brazzaville.[2] Moreover, travel to Kinshasa is frequent from Mbandaka. Given these risk factors, early epidemic growth profiles,[4] and evidence of unreported infection from previous outbreaks,[5,6] the risk of a substantially larger outbreak could not be ignored.

The factors causing epidemic growth to peak have been debated. Delayed detection of EVD outbreaks and resulting widespread distributions of EVD have significantly contributed to epidemic growth.[7] In addition to traditional burial practices, Ebola treatment units with low quality care and/or high mortality rates have discouraged Ebola suspects from presenting to care and contribute to community-based transmission.[8–10] Fragile, overwhelmed public health surveillance systems have also contributed to higher rates of unreported cases, who endanger urban communities,[11] which potentially have had higher transmission rates than rural communities.[12] Change to subcritical transmission (reproduction number below 1) tends to occur when Ebola response organizations deploy control, prevention and care measures,[13,14] communities adopt more protective behaviors,[15,16] and/or transmission decreases in a social network.[17,18] Scientific advances with rapid diagnostics and vaccines from the West Africa outbreak were deployed in the April-July 2018 EVD outbreak in DRC and had the potential to limit Ebola virus transmission.[19–21]

We used reported case counts during EVD outbreak in DRC and/or time series from 18 prior outbreaks to estimate the total outbreak size and duration with and without the use of vaccines. These projections were intended to help organizations anticipate and allocate sufficient resources for the duration of the April-July 2018 EVD outbreak.

## Methods

### Analysis

**Fig 1:**
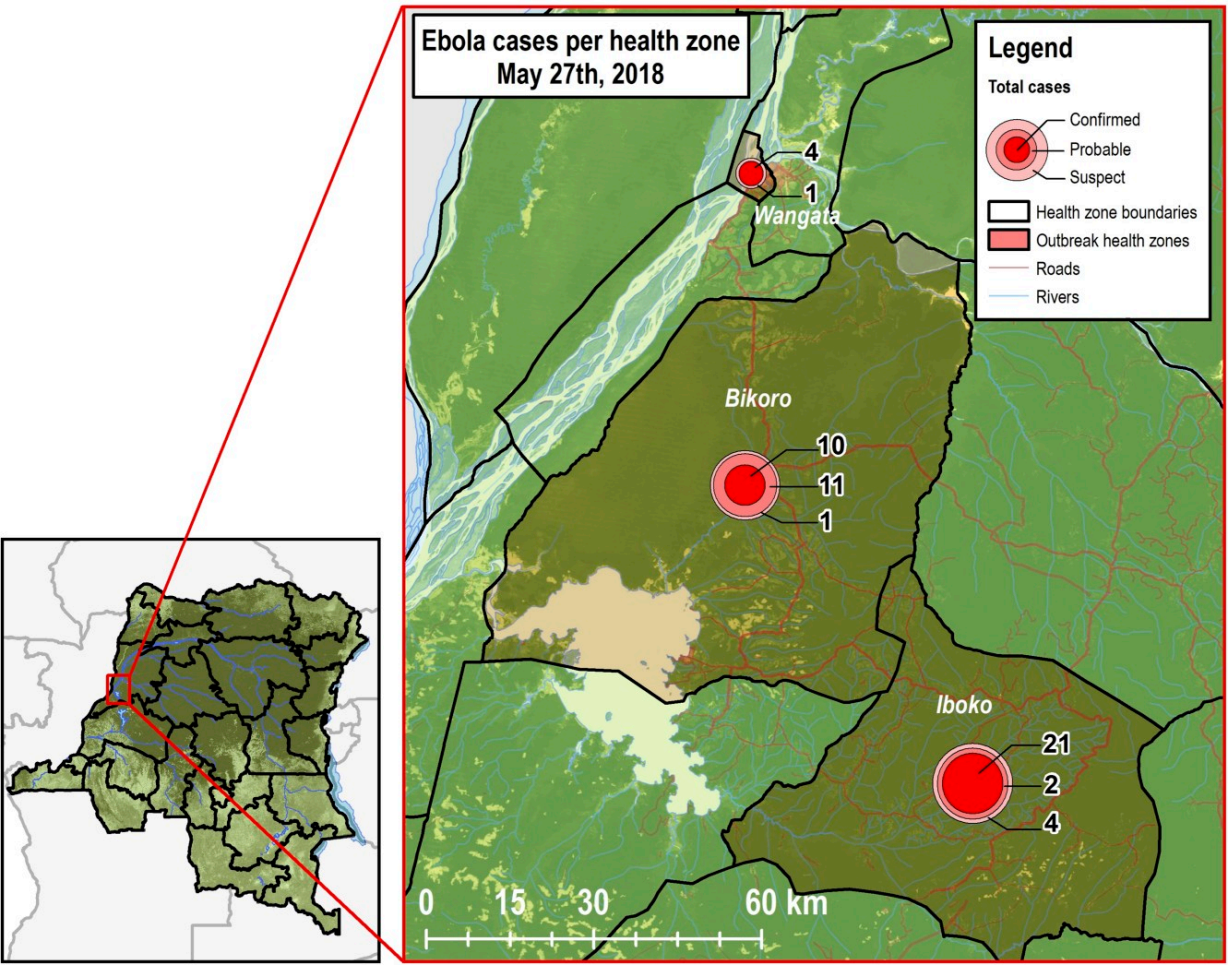
Map of cases of Ebola virus disease per health zone in Équateur Province, Democratic Republic of Congo, as of May 27, 2018 (Source: Hansen/UMD/Google/USGS/NASA[24]).

The following methods were used to generate projections: a stochastic branching process model,[22] statistical regression based on prior outbreaks, and Gott’s Law.[23] On May 27, 54 EVD cases (6 suspected, 13 probable and 35 confirmed) were reported in three locations (Iboko, Bikoro, and Mbandaka) (**Fig 1**). Based on EVD situation reports from DRC, we assumed the ring vaccination program started the week of May 21, so we used May 23 as the time point that vaccines were implemented in the model at high and low coverage levels.

### Data sources

Data on suspected, probable, and confirmed case counts were available from WHO situation reports in May 2018 and used as the basis for analysis. The published situation reports reflected data available up until May 11, 13, 16, 23, and 27, respectively. During the outbreak, the Ebola response team tested suspected cases for EVD and depending on positive or negative results, cases were classified as confirmed or not a case. The final outbreak case count was 54 probable and confirmed cases (no suspected cases).[1] Due to this reporting process, probable and confirmed cases were used to parameterize the stochastic branching process model, regression models, and Gott’s law. We added suspected cases to create an additional projection, using stochastic branching process model.

### Stochastic branching process model

We modeled Ebola virus transmission using a stochastic branching process model, parameterized by transmission rates estimated from the dynamics of prior EVD outbreaks, and conditioned on agreement with reported case counts from the 2018 EVD outbreak to date. We incorporated high and low estimates of vaccination coverage into this model. Then we generated a set of probabilistic projections of the size and duration of simulated outbreaks in the current setting.

To estimate the reproduction number *R* as a function of the number of days from the beginning of the outbreak, we included reported cases by date from thirteen prior outbreaks and excluded the first historical outbreak reported in those countries (e.g., 1976 outbreak in Yambuko, DRC) (**S1 Table**).[25–32] As there is a difference in the Ebola response system as well as community sensitization to EVD following a country’s first outbreak, we employed this inclusion criterion to reflect the Ebola response system in DRC during what is now its ninth outbreak. The Wallinga-Teunis technique was used to estimate *R* for each case and therefore for each reporting date in the outbreak.[33]

The serial interval was defined as the interval between disease onset in an index case and disease onset in a person infected by that index case. The serial interval distribution used for this estimation was a gamma distribution with a mean of 14.5 days and a standard deviation of 5 days, with intervals rounded to the nearest whole number of days, consistent with the understanding that the serial interval of EVD cases ranges from 3 to 36 days with mean 14 to 15 days.[11,34–36] Given that serial interval distribution, which we can denote as a probability *w(t)* of a *t*-day interval between given primary and secondary cases, Wallinga-Teunis estimation works by defining a relative likelihood *p*_*ij*_ for each possible source *j* of a given case *i*:

pij=w(ti–tj)/Σkw(ti–tk),

and then deriving from that an estimated reproduction number *Rj* for each case:

Rj=Σipij.

After using this technique to derive estimated reproduction numbers for each case in an outbreak, we use these estimated *R* values and cases’ onset dates *d* to estimate an initial reproduction number *R*_*0*_ and quenching rate τ for each past outbreak by fitting an exponentially quenched curve:

$$R=Ro\;e^{-\tau d},$$

to each outbreak's *R* and *d* values (**S1 Fig**).

Transmission was modeled using a stochastic branching process model in which the number of secondary cases *Si* caused by any given primary case *i* was drawn from a negative binomial distribution whose mean is the reproduction number *R*:[37,38]

Si∼Nb(R,k),

where *R* is reproduction number as a function of day, *k* is a dispersion parameter, and Nb() denotes the negative binomial distribution. All transmission events were assumed to be independent. The serial interval between date of detection of each primary case and that of each of its secondary cases is assumed gamma distributed with mean 14.5 days and standard deviation 5 days, rounded to the nearest whole number of days, as above. The pair of parameters *R*_*0*_ and τ estimated for the different past outbreaks used, and dispersion parameter *k*, were used in all possible combinations (with *R*_*0*_ and τ taken as a unit) to simulate outbreaks.

This model generated randomly varying simulated outbreaks with a range of case counts per day. After the Ministry of Health and WHO conducted epidemiological investigations about the beginning of the EVD outbreak in Équateur Province, they concluded that the outbreak began on April 4, 2018, with a single case.[1] The simulation process occurs as follows: proposed epidemic trajectories are generated in an initial step based on the above branching process, and these are then subsequently filtered by discarding all but those whose cumulative case counts match the known counts of the April-July 2018 EVD outbreak on known dates. The filtration accepts epidemics within a range of 3 cases more or less than each recorded value. This one-step particle filtering technique produced an ensemble of model outbreaks, filtered on agreement with the recorded trajectory of the outbreak to date. This filtered ensemble is then used to generate projections of the eventual outcome of the outbreak.[12]

To model vaccination coverage with respect to total transmission (unreported and reported), we multiplied the estimate of vaccine effectiveness by low and high estimates of reported cases. In a ring vaccination study at the end of the West Africa outbreak, the overall estimated rVSV-vectored vaccine efficacy was 100% and vaccine effectiveness was 64.6% in protecting all contacts and contacts of contacts from EVD in the randomized clusters, including unvaccinated cluster members.[20] Estimates of vaccine effectiveness were used in our stochastic model. The ring vaccination study found the vaccine to be effective against cases with onset dates 10 days or more from the date of vaccine administration, so we modeled the vaccination program as a proportionate reduction in the number of new cases with onsets 10 days or more after the program start date.

Then, past estimates of the proportion of unreported cases were used to estimate the proportion of exposed individuals not covered by the vaccination process. Based on a Sierra Leonean study from the 2013–2016 outbreak,[11] we estimated that the percentage of reported cases in DRC would rise over time from a low of 68% to a high of 96%. Given these low and high estimates of reported cases and the estimate of vaccine effectiveness (64.6%), a low estimate of vaccination program coverage was 44% (68% multiplied by 64.6%) and a high estimate of vaccination program coverage was 62% (96% multiplied by 64.6%). The course of the outbreak with and without the vaccination program was modeled based on approximate dates available from situation reports.[1]

For simulation based on probable and confirmed cases, from 122,683,392 simulated outbreaks, 21,036 were retained after filtering on approximate agreement with DRC case counts. For simulation based on probable, confirmed, and suspected cases, from 122,683,392 simulated outbreaks, 32,431 were retained after filtering. The simulated outbreaks that were retained after filtering were continued until they generated no further cases. This ensemble was used to derive a distribution of outbreak sizes and durations. Mean and median values and 95% prediction intervals were calculated using the 2.5 and 97.5 percentiles of simulated outbreak size and duration. These analyses were conducted using R 3.4.2 (R Foundation for Statistical Computing, Vienna, Austria).

### Regression model

For contrast with the stochastic model above, a simple regression forecast was conducted based solely on outbreaks of size 10 or greater. Time series for all 18 such prior outbreaks were obtained, including seven prior ones from DRC, dating back to 1976 (**S1 Table; S2 Fig**).[1,25–32,39–45] One (5.6%) of the 18 prior outbreaks in our sample (the 2013–2016 West Africa outbreak) achieved a large size (over 28,000 cases). No exclusions were made; no attempt to model specific features of the April-July 2018 outbreak was conducted. The regression model predicted the outbreak size based on values of the outbreak size at a specific earlier time. The beginning of each outbreak was not reliably characterized; therefore, all time-series were aligned on the day they reached 10 cases. In the April-July 2018 outbreak, we observed cases over the period from April 4 to May 27 (day 0 to day 53). May 27 corresponded to day 34 since reaching 10 reported cases. For the prior 18 outbreaks, linear interpolation was used to obtain the number of cases on day 34 (after reaching 10 cases). To reduce the influence of outliers and high leverage points, and to improve linearity, we calculated the pseudologarithm transform f(x) = arcsinh(x/2), asymptotically logarithmic but well-behaved at 0. We used non-parametric Theil-Sen regression (R-package *mblm*) followed by calculation of the resulting prediction interval for a new observation.[46,47] Finally, we reported the median and 95% central coverage intervals for the prediction distribution conditional on the value being no smaller than the observed value on day 34. Sensitivity analysis was conducted using ordinary least squares regression. These analyses were conducted using R 3.4.2 (R Foundation for Statistical Computing, Vienna, Austria).

### Gott’s Law

Gott’s Law was used to estimate the outbreak size using data through May 27 and May 11. We included a projection using data through May 11 because we hypothesized that this method performs better when the first situation report is posted than at later in the outbreak period.[23] Then we included a projection with the regression models using data through May 11 for comparison. With Gott’s Law, we assume we have no special knowledge of our position on the epidemic curve. If we assume a non-informative uniform prior for the portion of the epidemic that still remains, the resulting probability distribution for the remaining number of cases *y* is:

P(Y=y)=Yo/y2.

The median outbreak size was estimated, along with the two-sided 95% prediction interval.

### Likelihood scoring

Each of the above models assigned a probability to any possible value of the total outbreak size. The final outbreak size was 54 probable and confirmed cases, so we identified the probability of this equivalent number (53) from each model, as of May 27. Probabilistic projections were scored using a log-likelihood (ignorance) score.[48]

## Results

As of May 27, 2018, there were 6 suspected, 13 probable and 35 confirmed EVD cases. Bikoko had ten confirmed cases, 11 probable cases, and one suspected case. Iboko had 21 confirmed cases, two probable cases, and one suspected case. Mbandaka had four confirmed cases and one suspected case (**Fig 1**).

**Fig 2:**
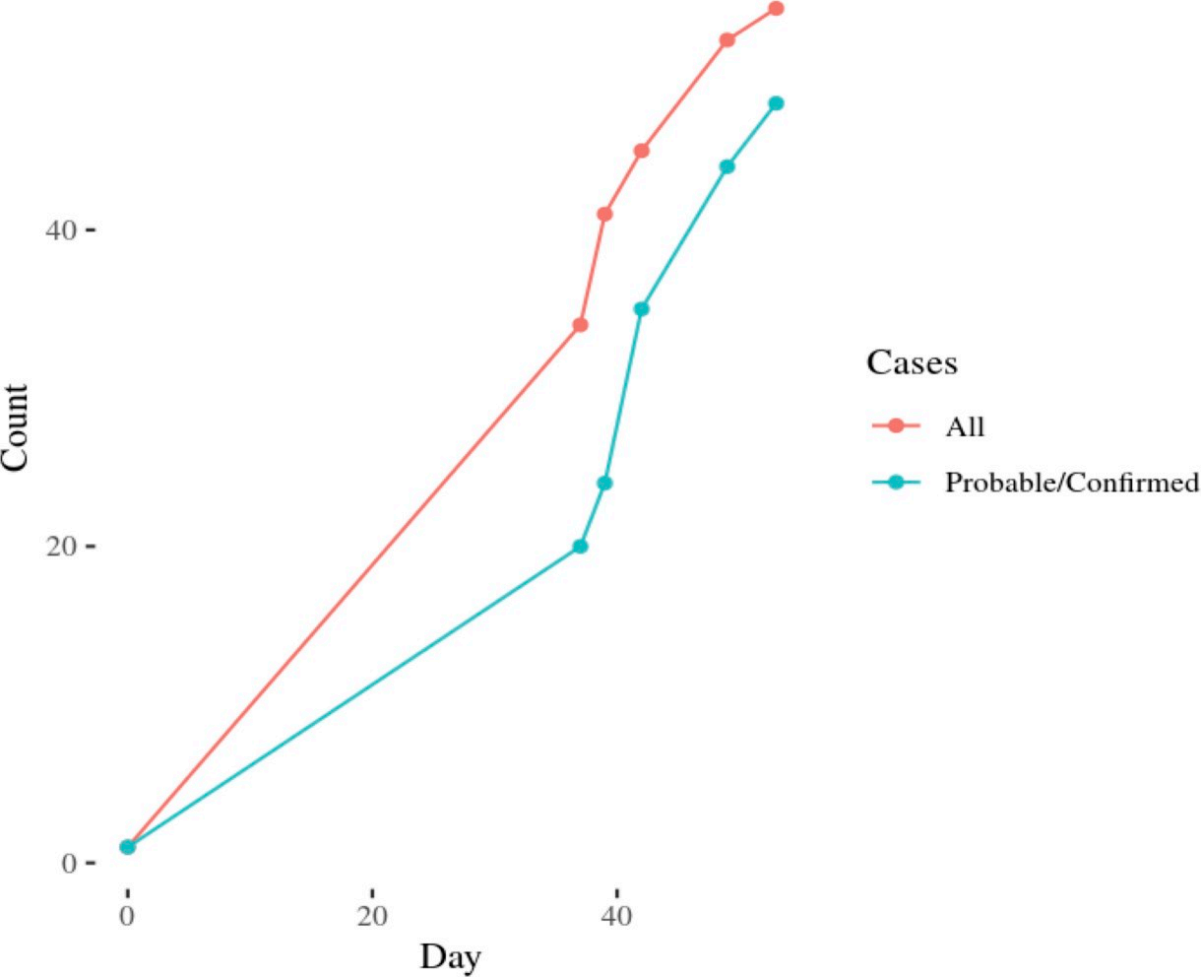
Cumulative case counts used in filtering stochastic branching process model.

**Fig 3:**
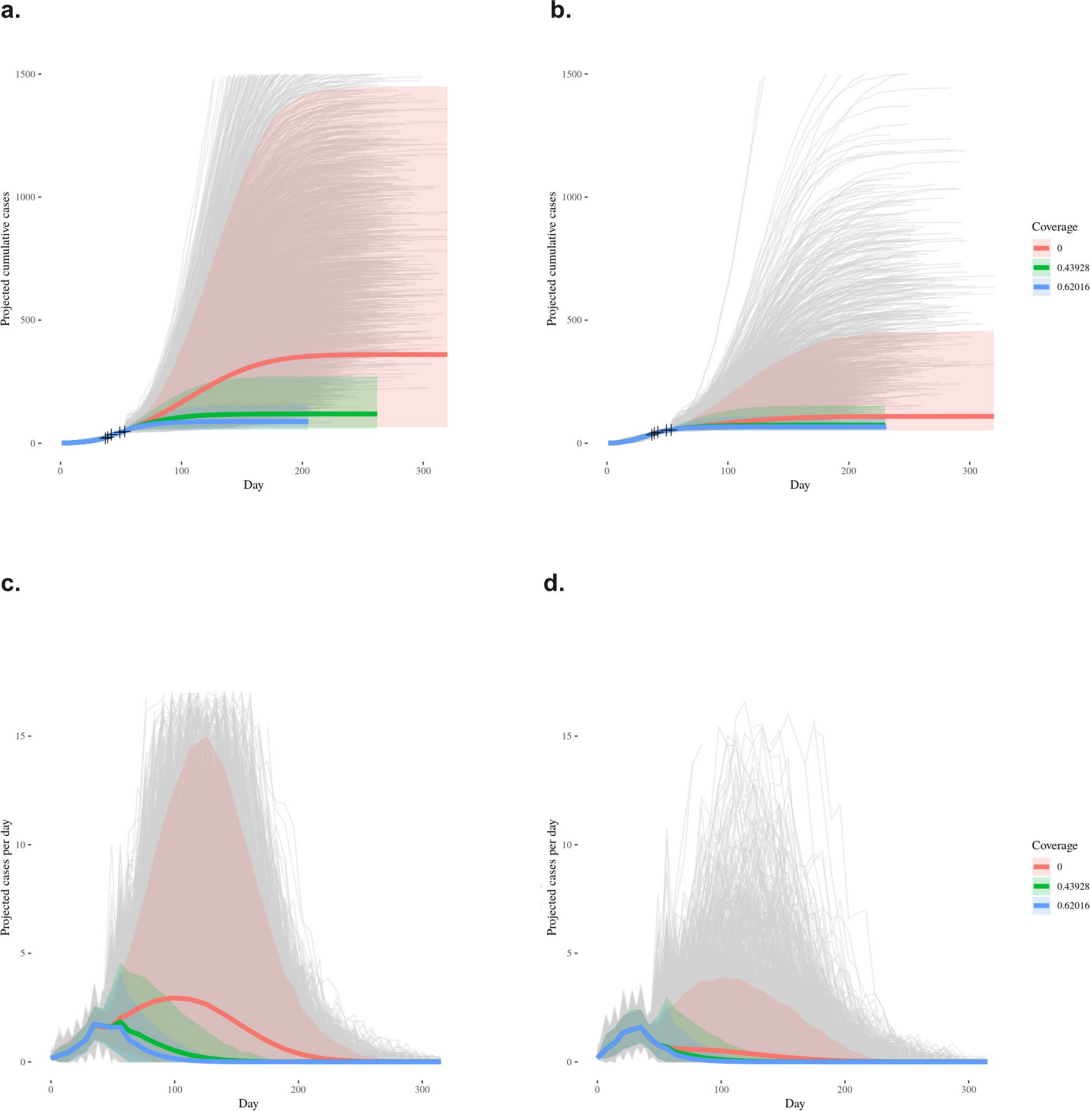
Distribution of outbreak projections from stochastic branching process model. **a.** Mean and prediction interval of cumulative **probable/confirmed** case count by day in model projections, by proportion of vaccine coverage. Target case numbers and dates are marked with + signs; **b.** Mean and prediction interval of cumulative **probable/confirmed/suspected** case count by day in model projections, by proportion of vaccine coverage. Target case numbers and dates are marked with + signs; **c.** Mean and prediction interval of weekly average of per-day **probable/confirmed** case count in model projections; and **d.** Mean and prediction interval of weekly average of per-day **probable/confirmed/suspected** case count in model projections.

With the stochastic model, we projected outbreak size and duration of probable and confirmed cases. In the absence of any vaccination program, the projected median outbreak size was 213.0 cases (mean 360.2; 95% prediction interval: 64.8, 1450.2). Median duration of projected outbreaks was 175.0 days (mean 182.8; 95% prediction interval: 86.0, 282.0). Using a lower estimate of 44% vaccination coverage, the median outbreak size was 104.0 cases (mean 118.8; 95% prediction interval: 58.0, 271.0) and median duration was 121.0 days (mean 124.6; 95% prediction interval: 74.0, 187.0). Using a higher estimate of 62% vaccination coverage, the median size was 82.0 EVD cases (mean 88.1; 95% prediction interval: 55.0, 156.0), and the median duration was 101.0 days (mean 103.4; 95% prediction interval: 68.0, 153.0). These projections with the stochastic model were repeated to estimate suspected, probable and confirmed cases **(Table 1 and Table 2; Figs 2 & 3)**.

**Table 1:** Distribution of projected outbreak size from stochastic branching process model. Mean, median and 95% prediction interval of outbreak size, by proportion of vaccine coverage, using probable and confirmed cases with and without suspected cases.

Cases	Vaccine Coverage	Median Size	Mean Size	95% Prediction Interval
Probable and confirmed	0	213.0	360.2	(64.8, 1450.2)
Probable and confirmed	0.44	104.0	118.8	(58.0, 271.0)
Probable and confirmed	0.62	82.0	88.1	(55.0, 156.0)
All	0	63.0	108.9	(51.0, 450.0)
All	0.44	62.0	74.0	(51.0, 153.0)
All	0.62	61.0	66.8	(51.0, 111.9)

**Table 2:** Distribution of projected outbreak duration from stochastic branching process model. Mean, median and 95% prediction interval of outbreak duration, by proportion of vaccine coverage, using probable and confirmed cases with and without suspected cases.

Cases	Vaccine Coverage	Median Duration	Mean Duration	95% Prediction Interval
Probable and confirmed	0	175.0	182.8	(86.0, 282.0)
Probable and confirmed	0.44	121.0	124.6	(74.0, 187.0)
Probable and confirmed	0.62	101.0	103.4	(68.0, 153.0)
All	0	78.0	101.1	(51.0, 241.0)
All	0.44	74.0	83.5	(51.0, 157.0)
All	0.62	70.0	76.0	(51.0, 129.0)

With the regression based on past outbreaks, the median outbreak size was estimated to be 65.0 probable and confirmed cases (95% prediction interval: 48.8, 119.7), while use of ordinary least squares produced a median size of 97.9 probable and confirmed cases (95% prediction interval: 58.3, 169.9). Outbreak projections were also reported using data through May 11 in **Table 3**.

**Table 3:** Distribution of outbreak projections from regression model. Median and 95% prediction interval of outbreak size, by date from which cases are predicted, and by regression method, using probable and confirmed cases. Method 1: Theil-Sen regression with a pseudo-log transformation. Method 2: Ordinary Least Squares (OLS) Regression without transformation.

	Using Data Through	Suspect Cases Included? (Y/N)	Total Outbreak Size: Median	95% Prediction Interval
Theil-Sen	May 27	N	65.0	(48.8, 119.7)
OLS	May 27	N	97.9	(58.3, 169.9)
Theil-Sen	May 11	N	27.7	(20.3, 52.5)
OLS	May 11	N	50.1	(28.5, 89.0)

Gott’s Law suggests that given 48 probable and confirmed cases, the median estimate of outbreak size was 96.0 cases (95% CI: 49.0, 1920.0). Using the 34 probable and confirmed cases as of May 11, the median estimate of outbreak size was 68.0 cases (95% CI: 35.0, 1360.0).

Of the mathematical models employed, the stochastic model that included suspected cases and high vaccination coverage had the best probabilistic score (log likelihood of -1.31). Likelihood scores of each model can be found in **Table 4**.

**Table 4:** Probabilistic scoring for stochastic branching process model, regression models and Gott’s Law. Date of prediction, vaccine coverage, inclusion of suspected cases and log-likelihood. For the regression model: Method 1—Theil Sen regression with a pseudo-log transformation. Method 2—OLS Linear Regression without transformation. Our mathematical models were scored against the actual outbreak size of 54 EVD cases. Note: “-”indicates that the model did not include vaccine coverage.

	Using Data Through	Vaccine Coverage	Suspect Cases Included? (Y/N)	Log Likelihood
**Stochastic**				
	May 27	0	Y	-1.98
	May 27	0.44	Y	-1.50
	May 27	0.62	Y	-1.31
	May 27	0	N	-2.92
	May 27	0.44	N	-2.53
	May 27	0.62	N	-2.27
**Regression**				
Theil-Sen	May 27	-	N	-3.47
Linear	May 27	-	N	-6.07
Theil-Sen	May 11	-	N	-5.92
Linear	May 11	-	N	-3.62
**Gott’s Law**				
	May 27	-	N	-4.07
	May 11	-	N	-4.97

## Discussion

When we were conducting our projections in late May, this outbreak still had the potential to become the largest outbreak in DRC since 2007. Vaccine use, regardless of coverage levels, was projected to prevent more than half of the total outbreak size. Vaccines, however, were only part of concurrent prevention, control, and care strategies.[8,49–51] We also found that the stochastic model with vaccine use projected that rare, large outbreaks (tail of the distribution of the model without vaccinations) were prevented, suggesting that repeat epidemics such as the 2013–2016 West African outbreak may have been highly unlikely once vaccines were rolled out.

Multiple models were used to estimate total outbreak size. This study exemplified how mathematical models, including simple regressions, can be useful for advising real-time decision making because they provided rapid projections and similar estimates of *R* as compared to complex models,[35] even though real-time modeling projections historically overestimated outbreak size and duration.[52,53] Our projections that included suspected cases did not suggest that vaccines had as much of an impact as our model using only probable and confirmed cases. The trends associated with suspected cases were subject to several factors, including operational choices of response teams and maturity of the outbreak. Nevertheless, suspected case counts may at times provide a better glimpse into the near future of an outbreak than the confirmed and probable case counts. In our case, use of the time series of confirmed, probable, and suspect cases yielded a forecast closer to the final outbreak size. However, as model projections can be highly sensitive to inclusion of suspected cases and use of exact case counts, particularly the last few counts in the available data, conclusions must be taken with caution.

Thus far, there had been a strong local and international response, and deployment of vaccines and rapid diagnostic tests (RDT) occurred early in response efforts.[1] RDTs were being used to screen Ebola suspects while the vaccines are being administered to high-risk groups for EVD, including healthcare workers, contacts, and contacts of contacts. To further limit epidemic growth from unreported cases, particularly those who have non-specific symptoms but are screened negative by the WHO case definition, more decentralized use of RDTs should be considered. In addition, formal evaluations of vaccine use and coverage on transmission reduction are needed.

There are limitations to our projections. Projection distributions were right-skewed, with long tails (and we therefore reported the median instead of the mean). While there have been 22 observed EVD outbreaks with a case count greater than ten cases, we were unable to include all prior outbreaks in our estimates due to data availability.[45,54] Note that the simple regression projection is based entirely on past outbreaks of EVD (measured and reported in different ways), and cannot account for the improved control measures and vaccination in the way that a mechanistic model does. We included, however, as much real-time information into our estimates as possible, but situations such as the introduction of EVD into a large urban population and implementation of RDTs and vaccines are new to DRC. We did not include vaccination of healthcare workers in the stochastic model. Our estimates of vaccination effectiveness and reported cases were obtained from West Africa because these estimates were not available for the EVD outbreak in Équateur. These modeling assumptions may not have been consistent with estimates in DRC and should be carefully considered prior to use in other EVD outbreaks.

A strength of our approach was the use of multiple methods to estimate the outbreak size, although we note that Gott’s Law has not been validated for outbreak projections in other EVD outbreaks. Additional limitations of the models were that they did not include parameters to address spatial spread, urban settings, conflict zone, or other factors that may have influenced the accuracy of the predictions, particularly in the 2018–2019 EVD outbreak in Northeastern DRC (ongoing in January 2019). While it can also be useful and achievable to use models of these kinds to make short-term forecasts for evaluation of model performance and to inform outbreak response,[55] the present study was limited to projections of final outbreak size and duration.

Among our three mathematical models, the model that performed the best (stochastic model with suspected cases and high vaccine coverage) predicted total outbreak sizes close to the true outcome. When EVD cases were introduced into Mbandaka, there was concern that the total outbreak size could exceed most prior EVD outbreaks in DRC. Indeed, our projections were consistent with this concern because models without vaccine coverage projected higher total outbreak sizes. In our stochastic model projections, vaccine use reduced mean total outbreak size by more than half, regardless of coverage levels (*p*<0.001, Welch’s *t*-test). As vaccine coverage was scaled up, an influx of support was warranted to support and bolster the evolving rapid response; however, continued efforts to strengthen the health system are equally as warranted so that we can respond to future outbreaks before they become epidemics. Relatively simple mathematical models updated in real time may inform outbreak response teams with projections of total outbreak size and duration.

## Supporting information

S1 Table List of 21 prior Ebola outbreaks from 1976 to 2016 by time period, country, confirmed/probable reported and time series case count, outbreak inclusion into the regression and stochastic models. (DOCX)

S1 Fig Estimates of temporal change in reproduction number *R* in thirteen past Ebola outbreaks. To each series of *R* estimates we fit an exponentially decaying curve, to be used in our simulations.
(TIFF)

S2 Fig Time-series of 18 prior Ebola outbreaks from 1976 to 2016 was depicted. (TIFF)
