# Supplementary material for: Projections of Ebola outbreak size and duration with and without vaccine use in Équateur, Democratic Republic of Congo, as of May 27, 2018
Source: PLoS One. 2019 Mar 7;14(3):e0213190. doi: 10.1371/journal.pone.0213190 (PMC6405095; doi:10.1371/journal.pone.0213190)
Supplement: S1 Table — (DOCX) [file pone.0213190.s001.docx]

**S1 Table. List of 21 prior Ebola outbreaks from 1976 to 2016** by time period, country, confirmed/probable reported and time series case count, outbreak inclusion into the regression and stochastic models.

| **Time Period** | **Country** | **Reported Case Count** | **Time Series Case Count** | **Regression?** | **Stochastic Model?** |
| --- | --- | --- | --- | --- | --- |
| Aug-Sep 1976 | DRC* | 318 | 262 | Yes | No |
| Jun–Nov 1976 | Sudan | 284 | 284 | Yes | No |
| Aug–Sep 1979 | Sudan | 34 | 34 | Yes | Yes |
| Dec 1994–Feb 1995 | Gabon | 52 | 49 | Yes | No |
| May–Jul 1995 | DRC | 315 | 317 | Yes | Yes |
| Jan–Apr 1996 | Gabon | 37 | 29 | Yes | Yes |
| Jul 1996–Mar 1997 | Gabon | 60 | – | No | No |
| Oct 2000–Jan 2001 | Uganda | 425 | 436 | Yes | No |
| Oct 2001–Jul 2002 | Gabon, Republic of the Congo | 124 | 119 | Yes | Yes |
| Dec 2002–Mar 2003 | Republic of the Congo | 143 | – | No | No |
| Nov–Dec 2003 | Republic of the Congo | 35 | 35 | Yes | Yes |
| Apr–Jun 2004 | Sudan | 17 | 17 | Yes | Yes |
| Apr–May 2005 | DRC | 12 | 12 | Yes | Yes |
| Aug–Nov 2007 | DRC | 264 | 264 | Yes | Yes |
| Dec 2007–Jan 2008 | Uganda | 131 | 127 | Yes | Yes |
| Dec 2008–Feb 2009 | DRC) | 32 | 32 | Yes | Yes |
| Jun–Aug 2012 | Uganda | 24 | 24 | Yes | Yes |
| Jun–Nov 2012 | DRC | 52 | 52 | Yes | Yes |
| Aug–Nov 2014 | DRC | 66 | 62 | Yes | Yes |
| Jul–Oct 2014 | Nigeria (offshoot of West African outbreak) | 20 | – | No | No |
| Jan 2014-Jun 2016 | Guinea, Liberia, Sierra Leone | 28,616 | 21,422 | Yes | No |

*Democratic Republic of Congo (formerly Zaire)
