# Supplementary figures and images for: Projections of Ebola outbreak size and duration with and without vaccine use in Équateur, Democratic Republic of Congo, as of May 27, 2018
Source: PLoS One. 2019 Mar 7;14(3):e0213190. doi: 10.1371/journal.pone.0213190 (PMC6405095; doi:10.1371/journal.pone.0213190)

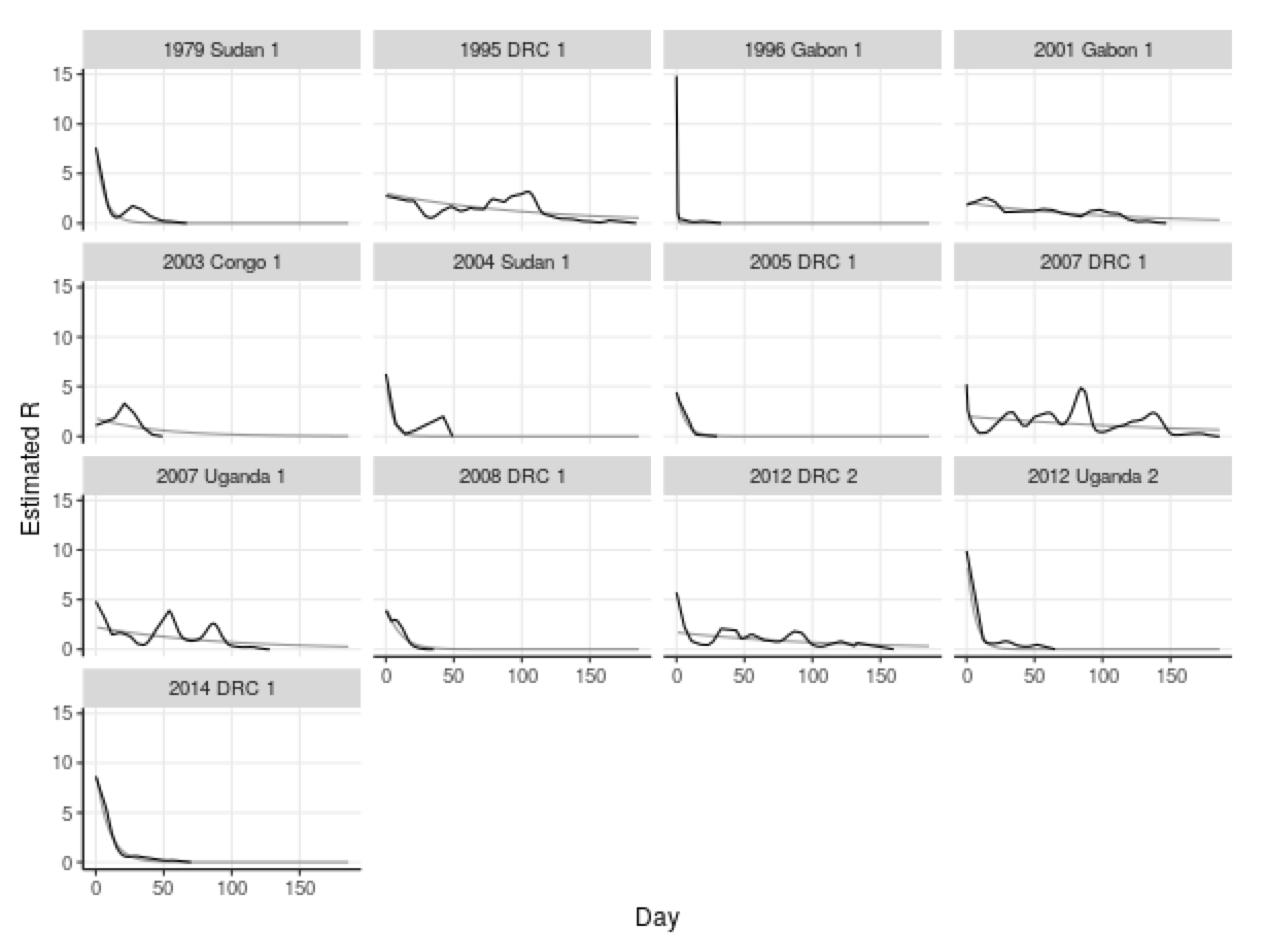

Supplement: S1 Fig — To each series of R estimates we fit an exponentially decaying curve, to be used in our simulations. (TIFF) [file pone.0213190.s002.tiff]

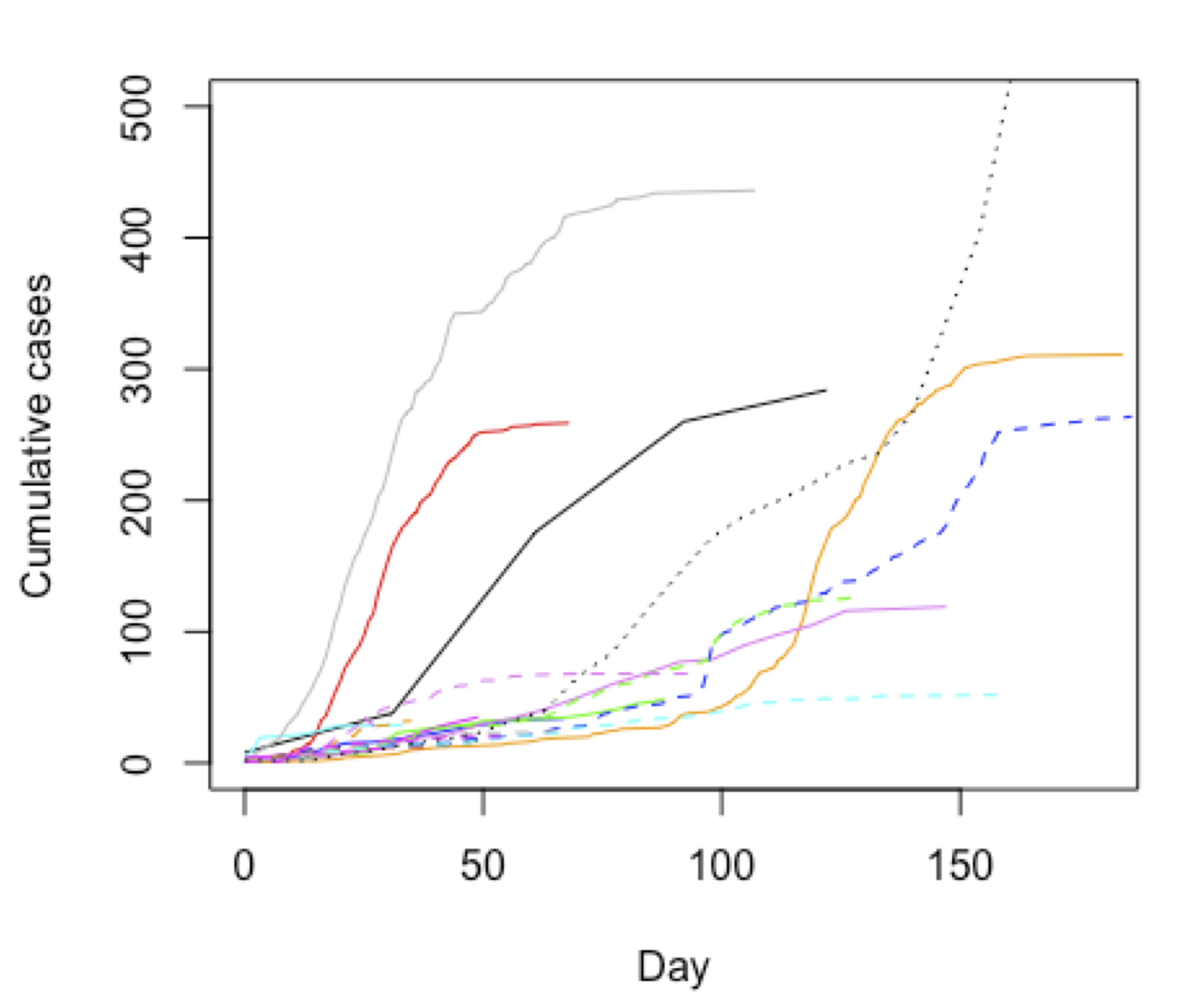

Supplement: S2 Fig — (TIFF) [file pone.0213190.s003.tiff]
